# Tumour necrosis factor-alpha (TNFα) stimulates the growth of human bone marrow stromal cells

**DOI:** 10.1080/09629359791730

**Published:** 1997-06

**Authors:** F. Rougier, E. Cornu, N. Gachard, V. Praloran, Y. Denizot

**Affiliations:** 1 Laboratoire d'Hématologie Expérimentale Faculté de Médecine 2 rue Dr. Marcland Limoges 87025 France; 2 Départment de Chirurgie Cardiothoracique CHRU Dupuytren Limoges 87042 France

## Abstract

This study reports that TNF-α is a potent mitogen for human bone marrow sternal cells *in vitro* (assessed by [^3^H]-thymidine incorporation into DNA and cell counts). In contrast, cytokines such as IL-1α, IL-1β, IL-2, IL-3, IL-4, IL-6, LIF, SCF, M-CSF, G-CSF and GM-CSF had no effect. The effect of TNF-α on the growth of human bone marrow stromal cells could be of importance during inflammatory processes which take place in the marrow, for example marrow fibrosis.


Mediators of Inflammation, 6, 233–235 (1997)

### Tumour necrosis factor-alpha (TNF-α) stimulates the growth of human bone marrow stromal cells

F. Rougier,[1] E. Cornu,[2] N. Gachard,[1] V. Praloran[1] and Y. Denizot[1,CA]

[1]Laboratoire d'Hématologie Expérimentale, Faculté de Médecine, 2 rue Dr. Marcland, 87025 Limoges, France; [2]Départment de Chirurgie Cardiothoracique, CHRU Dupuytren, 87042 Limoges, France

[CA]Corresponding author
Tel: (+33) 05 55 43 58 67
Fax: (+33) 05 55 43 58 01, (+33) 05 55 43 58 66

**This study reports that TNF-α is a potent mitogen for human bone marrow sternal cells *in vitro* (assessed by [$^3$H]-thymidine incorporation into DNA and cell counts). In contrast, cytokines such as IL-1α, IL-1β, IL-2, IL-3, IL-4, IL-6, LIF, SCF, M-CSF, G-CSF and GM-CSF had no effect. The effect of TNF-α on the growth of human bone marrow stromal cells could be of importance during inflammatory processes which take place in the marrow, for example marrow fibrosis.**

**Key words:** Cytokines, Growth, Marrow stromal cells, Tumour necrosis factor

### Introduction

Bone marrow stromal cells (mostly fibroblast-like cells) regulate hematopoiesis by producing cytokines and colony-stimulating factors (CSFs).[1,2] While numerous studies highlight the effects of stromal cell-derived cytokines on the growth of human hematopoietic progenitors,[3,4] less information is available on the role of cytokines and CSFs on the growth of marrow stromal cells. Tumour necrosis factor alpha (TNF-α) is a pleiotropic cytokine which mediates numerous biological responses such as immunomodulation, inflammation and antimicrobial defence.[5] Among its effects, TNF-α plays a role in the regulation of myelopoiesis, erythropoiesis, lymphopoiesis and thrombocytopoiesis.[6] since TNF-α is a mitogen for human dermal, foreskin and lung fibroblasts,[7–9] we have investigated its effect on human bone marrow stromal cell proliferation.

### Materials and Methods

This study was performed according to the Helsinki recommendations. Cultures of human bone marrow sternal cells were established from stromal bone marrow samples harvested from untreated patients referred for diagnosis. Mononuclear marrow cells were isolated by separation on a Ficoll gradient ($400 \times g$ 20 min), washed twice with HBSS ($400 \times g$, 10 min), and seeded in 75 cm$^2$ culture flasks ($2 \times 10^6$ cells/ml; $5 \times 10^5$ cells/cm$^2$) in RPMI 1640 with 20% FCS (Gibco, Cergy Pontoise, France), penicillin (100 U/ml) and streptomycin (100 μg/ml) (culture medium) at 37°C in 5% $CO_2$ in air as previously described.[10] After 1 week, non-adherent cells were removed from culture flasks. Adherent cells were grown to confluence for 3–4 weeks with weekly changes of medium and were subcultured after trypsin treatment. Cells were analysed on a Profile Coulter using Epics Profile Software to determine the cellular characteristics of the adherent layers. As previously reported,[11] more than 99.8% of cells were CD2$^-$ and CD22$^-$ indicating the absence of T and B cells on the layers and 4% of cells were CD14$^+$ and CD33$^+$ indicating a monocytic lineage.

Cells ($1 \times 10^4$/well) were plated for 24 hours in 96-well plates in 100 μl of culture medium. Adherent cells were washed with HBSS and 200 μl of serum-free medium was added to each well for 2 days. Adherent cells were reactivated with 100 μl of RPMI 1640 with 5% FCS and stimulated with TNF-α, interleukin-1α (IL-1α), IL-1β, IL-2 (Tebu, Le Perray en Yvelines, France), IL-3 (Sandoz, Rueil-Malmaison, France), IL-4 (Shering Plough Co.), IL-6, leukaemia inhibitory factor (LIF), stem cell factor (SCF) (Tebu), macrophage-CSF (M-CSF) (Cetus Corporation

© 1997 Rapid Science Publishers

Emeryville, CA), granulocyte-CSF (G-CSF) (Sandoz), and granulocyte–macrophage CSF (GM-CSF) (R & D Systems, Oxon, UK) or the appropriate vehicle (10 µl RPMI). After 60 hours of incubation, cultures (six replicates per sample) were pulsed for 12 hours with 1 µCi/ml [$^3$H]-thymidine and the cells were harvested using a Skatron cell harvester. In separate sets of experiments, cells (four replicates per sample) were harvested after trypsin treatment (0.05% trypsin for 5 min at 37°C) and counted by using a haemocytometer. In some experiments the effect of TNF-α was investigated with cells reactivated with 20% FCS and with cells grown in 5% FCS without the step of serum-deprivation. In other experiments TNF-α was added 24 hours after reactivation with 5% FCS.

Results were compared with the Mann–Whitney U-test or Student's t test for paired samples. A $P < 0{\cdot}05$ was considered significant.

### Results and Discussion

These experiments were done with marrow stromal cells from 25 different donors. Previous experiments showed that FCS increased [$^3$H]-thymidine incorporation by human bone marrow stromal cells in a dose-dependent manner with 5% FCS and 20% FCS as suboptimal and optimal concentrations, respectively.[2]

In growth synchronized cells, TNF-α at 1 ng/ml increased DNA synthesis by 230 ± 61% (Fig. 1). In similar experimental conditions (i.e., 5% FCS in growth medium), IL-1α, IL-1β, IL-2, IL-3, IL-4, IL-6, LIF, SCF, G-CSF, GM–CSF (both from 10 ng/ml to 0·1 ng/ml) and M-CSF (from 1000 UI/ml to 10 UI/ml) had no significant effect ($P > 0{\cdot}05$) on DNA synthesis (data not shown). The stimulatory effect of TNF-α at 1 ng/ml was found with cells from 15 of 15 different donors but was not observed with non-synchronized cells (data not shown). In growth medium with 20% FCS, TNF-α stimulated [$^3$H]-thymidine incorporation by 51 ± 9% (mean ± SEM of three experiments). Since the greatest increase was seen with 5% FCS in medium, we focused on this concentration for the remainder of the experiments.

We next assessed whether the observed stimulation of DNA synthesis was dependent on the time of TNF-α addition. When TNF-α was added 24 hours after reactivation with FCS, a significant ($P < 0{\cdot}05$, four experiments) increase in [$^3$H]-thymidine incorporation was found in TNF-α-treated cells (2552 ± 506 dpm) as compared with controls (1552 ± 522 dpm) indicating that serum-stimulated marrow stromal cells can be further regulated by TNF-α. [$^3$H]-thymidine uptake is considered a good index of cell proliferation but may also, in part, reflect intracellular events other than cell division such as difusion of DNA precursors.[12] Therefore, we assessed the effect of TNF-α on cell number. As shown in Fig. 1B, a significant increase of marrow stromal cell number was found 3 days after TNF-α stimulation.

This study indicates that numerous cytokines and growth factors, which act positively or negatively on the growth of human marrow progenitors, have no effect on the growth of human marrow stromal cells. Among the 12 molecules tested in this study, only TNF-α is a potent mitogenic stimulus for their growth. This

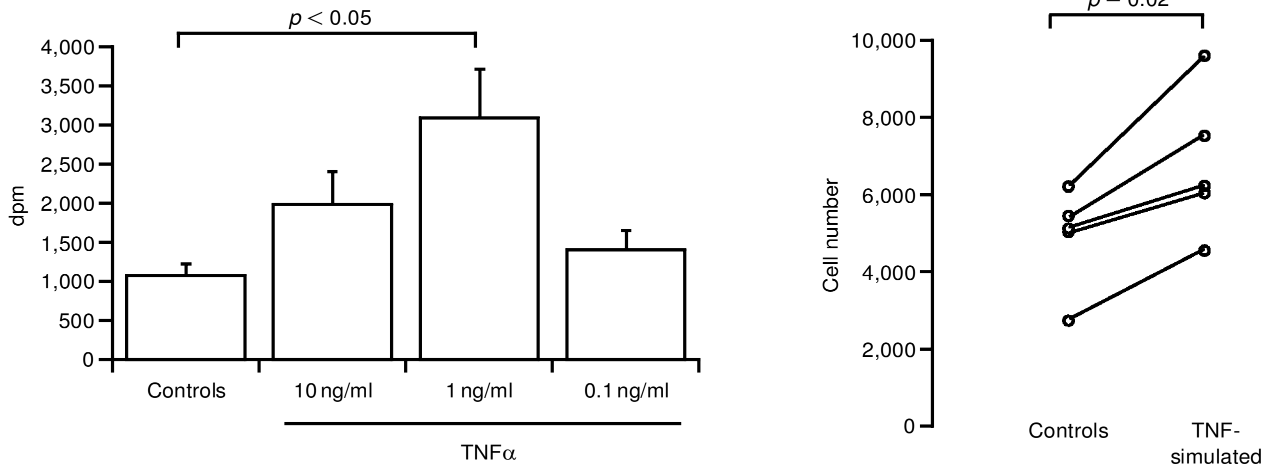

FIG. 1. Effects of TNF-α on the growth of human bone marrow stromal cells with 5% FCS in the culture medium. (A) Effects on [$^3$H]-thymidine incorporation: results (in dpm) are reported as mean ± SEM from four independent experiments each of six replicates. Data are compared with the Mann–Whitney U-test. (B) Effects on cell counts: experiments were performed with cells from five different donors. Data are compared with Student's t-test for paired data.

result agrees with the positive effect of TNF-α on the proliferation of human fibroblasts of different origins.[7–9] These results highlight for the first time a mechanism which could be of importance for understanding the increased population and activity of bone marrow fibroblasts in marrow fibrosis associated with several haematological malignancies.

### References

1. Mayani H, Guilbert LJ, Janowska-Wieczorek A. Biology of the hemopoietic microenvironment. *Eur J Haematol* 1995; **49**: 225–233.
2. Rougier F, Dupuis F, Denizot Y. Human bone marrow fibroblasts–an overview of their characterization, proliferation and inflammatory mediator production. *Hematol Cell Ther* 1996; **38**: 241–246.
3. Dorshkind K. Regulation of hemopoiesis by bone marrow stromal cells and their products. *Ann Rev Immunol* 1990; **8**: 111–137.
4. Moreau I, Duvert V, Caux C, *et al.* Myofibroblastic stromal cells isolated from human bone marrow induce the proliferation of both early myeloid and B-lymphoid cells. *Blood* 1993; **82**: 2396–2405.
5. Fiers W. Tumor necrosis factor. Characterization at the molecular, cellular and in vivo level. *FEBS Lett* 1991; **285**: 199–212.
6. Ulich TR, Shin SS, del Castillo J. Haematologic effects of TNF. *Res Immunol* 1993; **144**: 347–354.
7. Battegay EJ, Raines EW, Colbert T, Ross R. TNF-α stimulation of fibroblast proliferation. Dependence on platelet-derived growth factor (PDGF) secretion and alteration of PDGF receptor expression. *J Immunol* 1995; **154**: 6040–6047.
8. Cao X, Guy GR, Sukhatme VP, Tan YH. Regulation of the Egr-1 gene by tumor necrosis factor and interferons in primary human fibroblasts. *J Biol Chem* 1992; **267**: 1345–1349.
9. Siegel SA, Shealy DJ, Nakada MT, *et al.* The mouse/human chimeric monoclonal antibody cA2 neutralizes in vitro and protects transgenic mice from cachexia and TNF leathality in vivo. *Cytokine* 1995; **7**: 15–25.
10. Denizot Y, Rougier F, Dupuis F, *et al.* PAF and haematopoiesis. VIII. Biosynthesis and metabolism of platelet-activating factor by human bone marrow stromal cells. *Exp Hematol* 1996; **24**: 1327–1332.
11. Lorgeot V, Trimoreau F, Gachard N, Praloran V, Denizot Y. Leukemia inhibitory factor (LIF) concentrations in the bone marrow plasma of patients with hematologic malignancies. *Eur Cytokine Netw* 1977; **8**: 57–59.
12. Baud L, Perez J, Denis M, Ardaillou R. 1987. Modulation of fibroblast proliferation by sulfidopeptide leukotrienes: Effect of indomethacin. *J Immunol* 1987; **138**: 1190–1195.

ACKNOWLEDGEMENTS. We are grateful to the 'Ligue Nationale Contre le Cancer' (Comité de la Corrèze) and to 'Les artistes de la Gartempe' for funding our project.

**Received 12 March 1997; accepted 25 March 1997**
